# P.A.V.I.A. Study: Pervasiveness and Associated Factors of Video Slot Machine Use in a Large Sample of Italian Adolescents

**DOI:** 10.1007/s10899-024-10334-2

**Published:** 2024-07-22

**Authors:** Giansanto Mosconi, Paola Bertuccio, Ilaria Albertin, Marcello Esposito, Anna Polgatti, Franco Taverna, Diego Turcinovich, Sara Russo, Silvia Gaggi, Serena Barello, Andrea Amerio, Sabrina Molinaro, Silvano Gallus, Lorella Cecconami, Simone Feder, Tomaso Vecchi, Anna Odone

**Affiliations:** 1 School of Public Health, Department of Public Health, Experimental and Forensic Medicine, University of Pavia, Via Forlanini 2, Pavia, 27100 Italy; 2 Semi di Melo - Centre for Childhood and Adolescence Education and Research, Milan, Italy; 3 Faculty of Medicine and Surgery, University of Pavia, Pavia, Italy; 4 Department of Brain and Behavioral Sciences, University of Pavia, Pavia, Italy; 5 Department of Neuroscience, Rehabilitation, Ophthalmology, Genetics, Maternal and Child Health (DINOGMI), Section of Psychiatry, University of Genoa, Genoa, Italy; 6 IRCCS Ospedale Policlinico San Martino, Genoa, Italy; 7 Institute of Clinical Physiology, National Research Council of Italy, Pisa, Italy; 8 Department of Medical Epidemiology, Istituto di Ricerche Farmacologiche Mario Negri IRCCS, Milan, Italy; 9 Health Protection Agency of Pavia, Pavia, Italy; 10 IRCCS Mondino Foundation, Pavia, Italy; 11 Medical Direction, Fondazione IRCCS Policlinico San Matteo, Pavia, Italy

**Keywords:** Gambling, Slot machine, Adolescents, Minors, Teen-agers, Cross-sectional study

## Abstract

Video slot machines (VSM) are considered a particularly harmful gambling format; however, scant data is available on their use among underage Italian individuals. Two surveys were conducted in 2018 and 2022 involving 7,959 underage high school students (57.8% female) in Pavia, Northern Italy. We estimated adjusted odds ratios (aOR) and corresponding 95% confidence intervals (CI) for lifetime experience and current regular (at least monthly) use of VSM, according to family, educational and behavioral factors. Overall, participants reporting lifetime VSM experience were 13.2% (95% CI: 12.5 − 13.9), 15.2% (95% CI: 14.0–16.4%) in 2018, and 12.0% (95% CI: 11.1–13.0%) in 2022. Current regular VSM users were 1.4% (95% CI: 1.1–1.7) in total, 1.2% (95% CI: 0.8–1.6%) in 2018 and 1.5% (95% CI: 1.1–1.8%) in 2022. VSM lifetime experience and current regular use were significantly more frequent in males (aORs: 1.55 and 4.81, respectively), students who failed a year (aORs: 2.07 and 3.44), or with daily gambling parents/siblings (aORs: 2.83 and 4.86). Lifetime use of alcohol, tobacco, or illicit substances was significantly directly associated with lifetime VSM use (aORs between 2.64 and 4.75); monthly alcohol, tobacco, or illicit substances use was significantly directly associated with current regular VSM use (aORs between 4.47 and 18.21). Sexting and voluntary self-injury were significantly more frequent among VSM lifetime/current regular users. VSM use, which is directly associated with other risky behaviors, may be pervasive among Italian minors. Such public health concern calls for legislative enforcements and integrated multidisciplinary health promotion and prevention strategies.

**Supplementary Information:**

The online version contains supplementary material available at 10.1007/s10899-024-10334-2.

## Introduction

Starting from the late 1970s, traditional slot machines with physical mechanic reels were gradually replaced by software-based virtual versions that replicated the gambling experience on screen displays: video slot machines (VSM) (Dixon et al., 2010; Jensen et al., 2013; Schüll, 2014). At the time being, in Italy, VSMs are legally available, for adults only, on two categories of electronic gaming machines (EGMs) – namely, Amusement With Prize (AWPs) machines and Video Lottery Terminals (VLTs) – and on licensed online gambling platforms. Introduced in the country in 2004, AWPs, also known as “new slots”, can be found not only in gambling halls but also in public places like bars, tobacco shops, paper shops, and hotels (*Apparecchi da intrattenimento*, 2023; *Sisal. Gaming machine*, 2023). VLTs, which first appeared in 2009, permit higher-stake wagers and are exclusively installed in gambling halls (*Apparecchi da intrattenimento*, 2023; *Gaming machine*). In spite of the relatively low prevalence of users in the country (Lugo et al., 2021), the latest available data show that EGMs account for the largest part of the state’s treasury revenue collection from land-based gambling (*Libro blu 2021 - Principali performances*, 2021). Moreover, in Italy, VSMs, like any other casino game, are accessible on authorized online gambling platforms (*Gambling law and regulation in Italy | CMS Expert Guides*, 2023), which were made available in 2011 and have, since 2020, generated more tax revenue than land-based gambling (*Libro blu 2021 - Principali performances*, 2021).

Both land-based and online VSMs share a distinctive design that involves high event frequencies, near misses, random ratio reinforcement schedules, multi-line betting, losses appearing as winnings, as well as sound and visual reinforcements (Auer & Griffiths, 2022; Dixon et al., 2013; Livingstone, 2017; Yücel et al., 2018). Such structural characteristics are believed to to exert a significant influence on the user’s decision-making process and behavior, facilitating escalation to harmful gambling patterns (Murch & Clark, 2021; Shao et al., 2013; Yücel et al., 2018). Indeed, available evidence suggests their use has a stronger association with problem and disordered gambling compared to other gambling formats (Breen & Zimmerman, 2002; MacLaren, 2015; Mazar et al., 2020; Scalese et al., 2015).

To prevent underage use of AWPs and VLTs, Italy enacted a legislative decree in 2012 that prohibited the establishment of new slot halls and the installation of additional AWPs within a secure distance around designated “sensitive locations,” including schools and youth gathering places (Tavazzani et al., 2020). Furthermore, in 2018, the Italian government passed a law on the integration of an age verification system based on health card scanning in all VLTs, becoming fully effective in 2020 (Tavazzani et al., 2020). In Italy, access to legally authorized gambling platforms has always been subject to age verification through authentication of personal documents. Consequently, online VSM use should be limited to adults only, from a merely formal perspective (“LEGGE 7 luglio, 2009, n. 88 - Disposizioni per l’adempimento di obblighi derivanti dall’appartenenza dell’Italia alle Comunita’ europee - Legge comunitaria 2008. (09G0100),” 2009). However, the effectiveness of such restrictions remains a matter of debate (Armitage, 2021; Marionneau et al., 2022; Rolando et al., 2020).

While children and adolescent gambling is globally gaining recognition as a growing public health concern (Armitage, 2021; Calado et al., 2017; Messerlian et al., 2005), the absence of up-to-date observational studies on underage VSM use in Italy hinders the understanding of the phenomenon’s extent, associated factors, and the identification of potential areas for improvement in prevention strategies. Furthermore, given the potential impact of the COVID-19 pandemic on gambling patterns (Lugo et al., 2021), there is an urgent need for additional research on this topic to monitor recent developments.

During the past decade, Pavia, northern Italy, has become internationally known as the gambling capital of the country because of its dense concentration of AWPs and VLTs (Povoledo, 2013). Taking advantage of two large cross-sectional surveys conducted by the Semi di Melo Research Centre in 2018 and 2022 in Pavia’s high schools  (Feder et al., 2023), the present study aimed to gain insights into the usage patterns of both land-based and online VSMs, along with associated factors, among underage Italian students.

## Methods

### Study Design, Setting, and Population

A repeated cross-sectional study was carried out using data collected within two web-based surveys designed by the Semi di Melo Research Centre in the context of the “Selfie project” in Pavia, Lombardy Region, northern Italy, targeting high school students. This initiative aims to gather data on the lifestyles, social interactions, and mental well-being of Italian middle and high school students through periodic surveys conducted in several Italian cities, with the goal of raising awareness of potential risks during adolescence. A comprehensive description of the “Selfie project” has been provided elsewhere (Feder et al., 2023). The first survey was conducted between January and June 2018, while the second survey was conducted from January to June 2022. Overall, 11 High Schools were involved. After merging data collected from the two surveys, the final sample comprised 7,959 students aged 15–17 years. There was no overlap of participants between the two surveys.

### Data Collection

Students were given a clear explanation of the research purpose and invited to participate by completing a fully anonymous online questionnaire accessible on school computers. Written informed consent was obtained from all participants. Participation was voluntary and fully anonymous, contingent upon informed consent for data processing for research purposes through an electronic form. Data collection occurred during school hours, and no compensation was provided for participation. The project received approval from all included school boards and the University, and adhered to the Declaration of Helsinki at all stages. The questionnaire explored demographic characteristics, family context, educational background, gambling and other risky behaviors – including tobacco smoking, alcohol drinking, and psychotropic substance use – sexting, and voluntary self-injury. Regarding risky behaviors, sexting, and voluntary self-injury, information was collected on the participants’ lifetime experience, defined as any experience of such activities during the participants’ life. The questionnaire also assessed the frequency of current risky behaviors by asking participants to choose among the following options: “never”, “less than once a month”, “1 to 4 times a month”, “2 to 4 times a week”, and “every day or almost every day”.

Within a dedicated section on gambling, the survey explored lifetime experience, current use, frequency and method of participation in various gambling formats, including VSM (meaning Amusement With Prize, Video Lottery Terminals, and online slot machines). Additionally, the questionnaire inquired about parents or sibling engaging in gambling on a daily or nearly daily basis. Participants’ perceptions and beliefs regarding gambling accessibility and related negative consequences were also examined.

### Outcomes and Variables of Interest

We selected the following outcomes of interest: “lifetime experience of VSM use”, intended as any VSM ever use, and “current regular VSM use”, defined as reporting to use VSMs at least once a month. We opted to focus on monthly VSM use to identify habitual users and distinguish them from those who only occasionally experimented with VSM. This definition of regular users aligns with recent studies by other authors (Chóliz et al., 2022; Mazar et al., 2020), where monthly gambling frequency was used as a benchmark for regularity. We considered gender, family context (parents’ employment, cohabitants, daily or nearly daily gambling by parents or siblings), educational background (course failures, school year failures), lifetime experience and frequency of selected risky behaviors (tobacco smoking, alcohol drinking, and cannabis, cocaine or other psychotropic substances use), and lifetime experience of sexting and voluntary self-injury as potentially associated factors.

### Statistical Analysis

To investigate factors associated with lifetime experience of VSM use and current regular VSM use, we estimated both unadjusted (uOR) and adjusted odds ratios (aOR) and their corresponding 95% confidence intervals (CIs) through logistic regression models adjusted by year of interview (2018, 2022), gender (male, female), age (continuous), nationality (Italian, not Italian born in Italy, not Italian born abroad), and type of school attended (lyceum, technical college, vocational college). All statistical analyses were performed using the software SAS version 9.4 and R Studio version 4.1.1.

## Results

A total of 10,547 responses were collected over the two survey rounds and 7,959 (75.5%) subjects aged 15–17 were included in the analysis: 3,372 from the 2018 survey, 4,587 from the 2022 survey.

Table 1 reports the distribution of the study population by selected demographic, family, and educational characteristics. Overall, 55.8% of participants were females; most had Italian nationality (86.3%), were attending a Lyceum (47.5%), had never failed a school year or course (61.9%), had both parents working (63.5%), and cohabited with both parents and at least one sibling (59.8%). The distribution of the study population by demographic, family, and educational characteristics was similar in the two samples.

**Table 1:** Distribution of 7,959 high school students aged 15–17, by selected demographic, family, and educational characteristics. Pavia, Lombardy Region, Italy (2018–2022)

	2018		2022		Total	
	n	%	n	%	**n**	**%**
**Overall**	3,372	100.0	4,587	100.0	7,959	100.0
**Sex**						
Males	1,422	42.2	2,094	45.7	3,516	44.2
Females	1,950	57.8	2,493	54.3	4,443	55.8
**Nationality**						
Italian	2,944	87.3	3,924	85.5	6,868	86.3
Foreign (born in Italy)	197	5.8	443	9.7	640	8.0
Foreign (born abroad)	231	6.9	220	4.8	451	5.7
**Type of school attended**						
Lyceum	1,562	46.3	2,219	48.4	3,781	47.5
Technical college	1,037	30.8	1,429	31.2	2,466	31.0
Vocational college	773	22.9	939	20.5	1,712	21.5
**School performance**						
Never failed a year or a course	1,990	59.0	2,937	64.0	4,927	61.9
Never failed a year but failed a course	882	26.2	1,168	25.5	2,050	25.8
Failed a year	500	14.8	482	10.5	982	12.3
**Parents employment**						
Both parents working	2,097	62.2	2,959	64.5	5,056	63.5
One parent working	927	27.5	1,211	26.4	2,138	26.9
Other	348	10.3	417	9.1	765	9.6
**Household composition**						
Both parents plus siblings	2,008	59.5	2,749	59.9	4,757	59.8
Both parents and no siblings	673	20.0	995	21.7	1,668	21.0
One parent plus siblings	418	12.4	516	11.2	934	11.7
One parent and no siblings	273	8.1	327	7.1	600	7.5

**Fig. 1:**
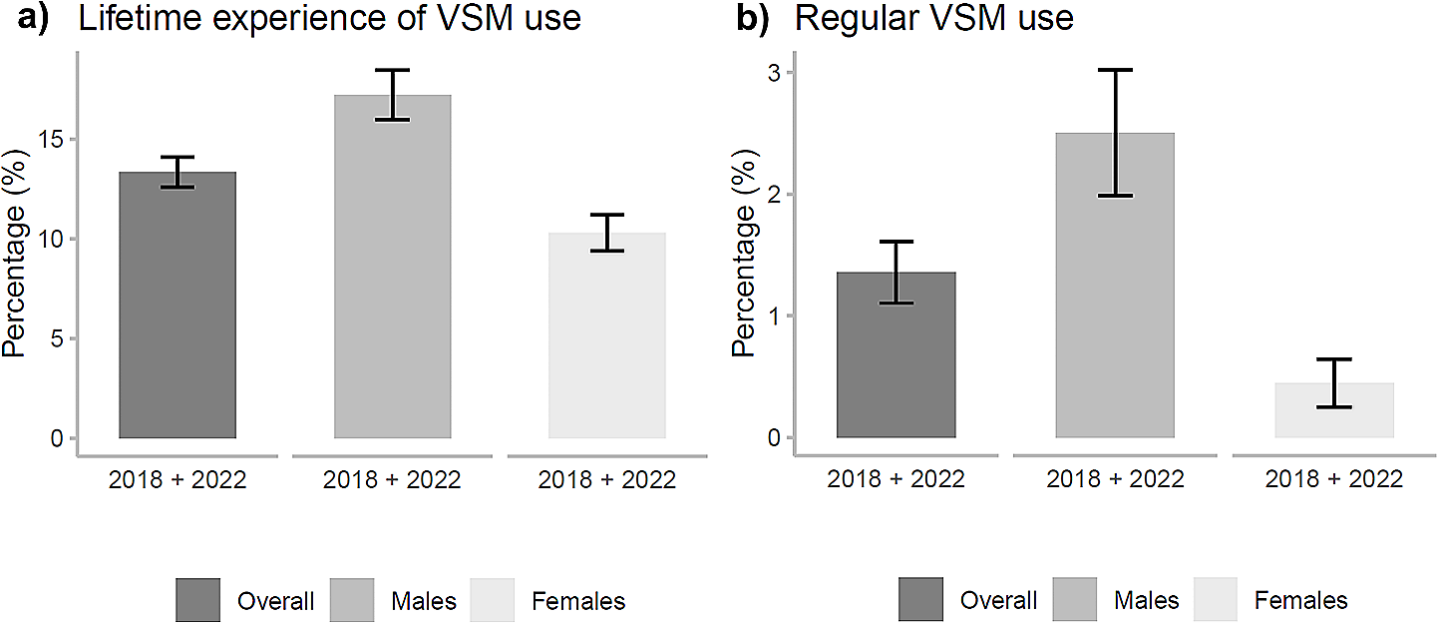
Overall and gender-specific proportion of subjects reporting (**a**) lifetime experience of video slot machine (VSM) use and (**b**) current regular VSM use among high school students aged 15–17. Pavia, Lombardy Region, Italy

**Fig. 2:**
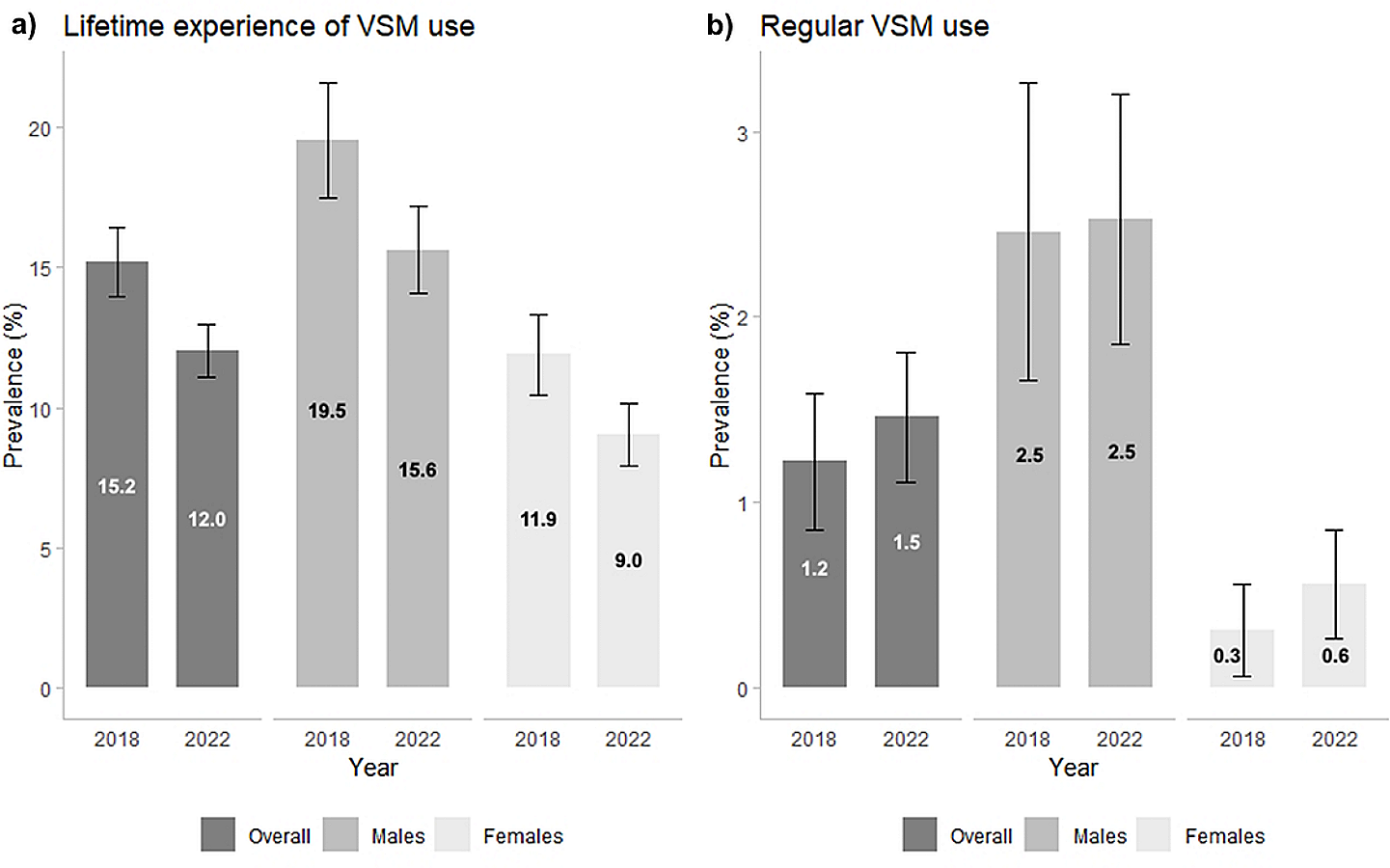
Overall and gender-specific proportion of subjects reporting (**a**) lifetime experience of video slot machine (VSM) use and (**b**) current regular VSM use among high school students aged 15–17 in 2018 and 2022. Pavia, Lombardy Region, Italy

In total, across both surveys, 13.2% (95% CI: 12.5–13.9) of participants reported lifetime experience of VSM, and 1.4% (95% CI: 1.1–1.7) reported current regular VSM use (Fig. 1a and b). The overall proportion of participants reporting lifetime VSM experience was 15.2% (95% CI: 14.0–16.4%) in 2018 and 12.0% (95% CI: 11.1–13.0%) in 2022 (*p*-value < 0.0001). Among males, the proportion was 19.5% in 2018 (95% CI: 17.5–21.6%) and 15.6% (95% CI: 14.1–17.2%) in 2022 (*p*-value: 0.0024), while among females 11.9% (95% CI: 10.5–13.3%) and 9.0% (95% CI: 7.9–10.2%), respectively (*p*-value < 0.01) (Fig. 2a). The overall proportion of current regular VSM users was 1.2% (95% CI: 0.8–1.6%) in 2018 and 1.5% (95% CI: 1.1–1.8%) in 2022 (*p*-value: 0.3510). The proportion was 2.5% (95% CI: 1.7–3.3%) in 2018 and 2.5% (95% CI: 1.9–3.2%) in 2022 among males (*p*-value: 0.8967), and 0.3% (95% CI: 0.1–0.6%) in 2018 and 0.6% (95% CI: 0.3–0.9%) in 2022 among females (*p*-value: 0.2097) (Fig. 2b).

In 2018, 24.4% of VSM current users declared using VSMs mainly in slot halls, 68.3% in bars and tobacco shops, and 2.4% on online gambling platforms. In 2022, 28.3% of current regular VSM users declared using VSMs predominantly in slot halls, 41.8% in bars and tobacco shops, and 22.4% on online gambling platforms.

Table 2 reports both uOr and aOR with corresponding 95% CIs for the association between lifetime experience of VSM use and gender, family, educational, and behavioral factors over the survey rounds. Males more frequently reported lifetime time experience of VSM use than females (aOR: 1.55, 95% CI: 1.36–1.78). As compared to participants who never failed a school year or course, lifetime experience of VSM use was reported twice more frequently by those who had failed a school year (aOR: 2.07, 95% CI: 1.71–2.45), and about 40% more frequently by those who had only failed a course in the past but never failed a school year (aOR: 1.42, 95% CI: 1.22–1.66). Regarding family context, compared to students cohabiting with both parents and at least one sibling, participants cohabiting with one parent more frequently reported lifetime experience of VSM use, both in the case they cohabited with at least one sibling (aOR: 1.37, 95% CI: 1.13–1.68) or not (aOR: 1.63, 95% CI: 1.30–2.05). Having a parent or sibling who gambled daily or almost daily was positively associated with lifetime experience of VSM use (aOR: 2.83, 95% CI: 2.38–3.37). Similarly, positive associations were found with lifetime experience of alcohol drinking (aOR: 3.27, 95% CI: 2.64–4.05), tobacco smoking (aOR: 2.64, 95% CI: 2.28–3.05), cannabis (aOR: 3.04, 95% CI: 2.64–3.49), cocaine (aOR: 4.75, 95% CI: 3.53–6.41) or other psychotropic substances (aOR: 3.76, 95% CI: 2.91–4.86) consumption. Additionally, a positive association emerged with having experienced sexting or voluntary self-injury (aOR: 2.41, 95% CI: 2.07–2.80 and aOR: 1.68, 95% CI: 1.46–1.94, respectively).

**Table 2:** Overall distribution (n) of 7,959 high school students aged 15–17 according to family, educational, and behavioral factors, and proportion of participants reporting lifetime experience of video slot machine (VSM)^#^ use, along with both unadjusted odds ratios (uOR) and adjusted^*^ odds ratios (aOR) and corresponding 95% confidence intervals (CI). Pavia, Lombardy Region, Italy (2018–2022)

			Lifetime experience of VSM use		
	*n*	%.		uOR* (95% CI)	aOR* (95% CI)
Total	7,959	13.2			
**Gender**					
Female	4,443	10.3		1.00°	1.00°
Male	3,516	17.2		**1.81 (1.59–2.07)**	**1.55 (1.36–1.78)**
**School performance**					
Never failed a year or course	4,927	10.7		1.00°	1.00°
Never failed a year but failed a course	2,050	15.1		**1.49 (1.28–1.73)**	**1.42 (1.22–1.66)**
Failed a year	982	23.2		**2.54 (2.13–3.02)**	**2.07 (1.71–2.45)**
**Cohabitants**					
Both parents plus siblings	4,757	12.1		1.00°	1.00°
Both parents and no siblings	1,668	13.3		1.11 (0.94–1.32)	1.15 (0.97–1.36)
One parent plus siblings	934	16.4		**1.42 (1.17–1.73)**	**1.37 (1.13–1.68)**
One parent and no siblings	600	18.5		**1.65 (1.32–2.06)**	**1.63 (1.30–2.05)**
**Parents’ employment status**					
Both parents working	5,056	12.7		1.00°	1.00°
One parent working	2,138	14.3		1.14 (0.99–1.33)	1.11 (0.95–1.29)
Other	765	14.6		1.18 (0.95–1.46)	1.06 (0.85–1.32)
**Parent or sibling gambling daily or almost daily**					
No	7,153	11.7		1.00°	1.00°
Yes	806	27.8		**2.90 (2.45–3.44)**	**2.83 (2.38–3.37)**
**Alcohol drinking**					
Never experienced	1,918	5.5		1.00°	1.00°
Already experienced	6,041	15.8		**3.25 (2.64–4.00)**	**3.27 (2.64–4.05)**
**Tobacco-smoking**					
Never experienced	3,947	7.9		1.00°	1.00°
Already experienced	4,012	18.7		**2.71 (2.35–3.11)**	**2.64 (2.28–3.05)**
**Cannabis consumption**					
Never experienced	5,950	9.2		1.00°	1.00°
Already experienced	2,009	25.5		**3.37 (2.95–3.86)**	**3.04 (2.64–3.49)**
**Cocaine consumption**					
Never experienced	7,763	12.6		1.00°	1.00°
Already experienced	196	43.9		**5.44 (4.07–7.27)**	**4.75 (3.53–6.41)**
**Other psychotropic substances consumption**					
Never experienced	7,671	12.5		1.00°	1.00°
Already experienced	288	36.8		**4.09 (3.19–5.25)**	**3.76 (2.91–4.86)**
**Sexting**					
Never experienced	6,595	11.3		1.00°	1.00°
Already experienced	1,364	23.5		**2.42 (2.09–2.80)**	**2.41 (2.07–2.80)**
**Voluntary self-injury**					
Never experienced	5,719	12.2		1.00°	1.00°
Already experienced	2,240	16.3		**1.41 (1.23–1.62)**	**1.68 (1.46–1.94)**

^#^ Video slot machines include: Amusement With Prize (AWPs) machines, Video Lottery Terminals (VLTs) and online slot machines

* Estimated through logistic regression models adjusted by year of interview (2018, 2022), gender (male, female), age (continuous), nationality (Italian, not Italian born in Italy, not Italian born abroad), and type of school attended (lyceum, technical college, vocational college). Estimates in bold are statistically significant at 0.05 level

° Reference category

Table 3 reports the estimates for the association between current regular VSM use and gender, family, educational, and behavioral factors over the two survey rounds. Males more frequently reported current regular VSM use than females (aOR: 4.81, 95% CI: 2.92–7.92). Students who had failed a school course or a year had higher odds for current regular VSM use compared to those who had not (aOR: 1.74, 95% CI: 1.08–2.81 and aOR: 3.44, 95% CI: 2.08–5.69, respectively). Students whose both parents were retired or unemployed more frequently reported current regular VSM use, than those whose parents were both employed at the time of the interview (aOR: 1.62, 95% CI: 0.96–2.74). A positive association was reported with having a parent or sibling who gambled daily or nearly daily (aOR: 4.86, 95% CI: 3.22–7.33). Similarly, there was a positive association with lifetime experience of alcohol drinking, tobacco smoking, cannabis, cocaine or other psychotropic substances consumption, sexting, and voluntary self-injury. Higher associations emerged among those who reported current alcohol drinking (aOR: 4.47, 95% CI: 2.85–7.00), tobacco smoking (aOR: 5.01, 95% CI: 3.36–7.48), and cannabis (aOR: 6.03, 95% CI: 4.08–8.93), cocaine (aOR: 18.21, 95% CI: 11.16–29.72), or other psychotropic substances use at least on a monthly basis (aOR: 16.81, 95% CI: 10.48–26.95), compared to those who did not.

**Table 3:** Overall distribution (n) of 7,959 high school students aged 15–17 according to gender, family, educational, and behavioral factors, and proportion of participants reporting current regular video slot machine (VSM)^#^ use, along with unadjusted odds ratios (uOR) and adjusted^*^ odds ratios (aOR) and corresponding 95% confidence intervals (CI). Pavia, Lombardy Region, Italy (2018–2022)

			Current regular VSM use		
	*n*	%.		uOR* (95% CI)	aOR* (95% CI)
Total	7,959	1.4			
**Gender**					
Female	4,443	0.5		1.00°	1.00°
Male	3,516	2.5		**5.68 (3.49–9.24)**	**4.81 (2.92–7.92)**
**School performance**					
Never failed a year or course	4,927	0.8		1.00°	1.00°
Never failed a year but failed a course	2,050	1.6		**1.94 (1.21–3.09)**	**1.74 (1.08–2.81)**
Failed a year	982	3.7		**4.65 (2.95–7.33)**	**3.44 (2.08–5.69)**
**Cohabitants**					
Both parents plus siblings	4,757	1.3		1.00°	1.00°
Both parents and no siblings	1,668	1.3		1.00 (0.61–1.62)	1.05 (0.64–1.72)
One parent plus siblings	934	1.3		0.97 (0.52–1.81)	0.95 (0.51–1.78)
One parent and no siblings	600	1.8		1.39 (0.73–2.66)	1.40 (0.73–2.70)
**Parents’ employment status**					
Both parents working	5,056	1.3		1.00°	1.00°
One parent working	2,138	1.0		0.77 (0.48–1.26)	0.73 (0.45–1.19)
Other	765	2.5		**1.90 (1.13–3.18)**	**1.62 (0.96–2.74)**
**Parent or sibling gambling daily or almost daily**					
No	7,153	1.0		1.00°	1.00°
Yes	806	4.7		**5.01 (3.35–7.48)**	**4.86 (3.22–7.33)**
**Alcohol drinking**					
Never experienced	1,918	0.6		1.00°	1.00°
Already experienced	6,041	1.6		**2.56 (1.40–4.68)**	**2.61 (1.41–4.81)**
**Alcohol drinking frequency**					
Never or less than once in a month	4,756	0.6		1.00°	1.00°
Monthly or more frequent	3,203	2.5		**4.33 (2.81–6.67)**	**4.47 (2.85–7.00)**
**Tobacco-smoking**					
Never experienced	3,947	0.6		1.00°	1.00°
Already experienced	4,012	2.1		**3.49 (2.22–5.51)**	**3.56 (2.23–5.67)**
**Tobacco-smoking frequency**					
Never or less than once in a month	5,955	0.7		1.00°	1.00°
Monthly or more frequent	2,004	3.2		**4.61 (3.13–6.80)**	**5.01 (3.36–7.48)**
**Cannabis consumption**					
Never experienced	5,950	0.6		1.00°	1.00°
Already experienced	2,009	3.6		**6.11 (4.08–9.14)**	**5.18 (3.41–7.87)**
**Cannabis consumption frequency**					
Never or less than once in a month	7,015	0.8		1.00°	1.00°
Monthly or more frequent	944	5.5		**7.25 (4.94–10.64)**	**6.03 (4.08–8.93)**
**Cocaine consumption**					
Never experienced	7,763	1.0		1.00°	1.00°
Already experienced	196	14.8		**16.89 (10.75–26.55)**	**13.22 (8.29–21.08)**
**Cocaine consumption frequency**					
Never or less than once in a month	7,818	1.0		1.00°	1.00°
Monthly or more frequent	141	19.1		**22.62 (14.09–36.31)**	**18.21 (11.16–29.72)**
**Other psychotropic substances consumption**					
Never experienced	7,671	1.1		1.00°	1.00°
Already experienced	288	9.4		**9.69 (6.16–15.24)**	**7.66 (4.82–12.17)**
**Other psychotropic substances consumption frequency**					
Never or less than once in a month	7,786	1.0		1.00°	1.00°
Monthly or more frequent	173	16.8		**19.65 (12.45–31.01)**	**16.81 (10.48–26.95)**
**Sexting**					
Never experienced	6,595	1.0		1.00°	1.00°
Already experienced	1,364	3.2		**3.27 (2.22–4.83)**	**3.32 (2.23–4.94)**
**Voluntary self-injury**					
Never experienced	5,719	1.2		1.00°	1.00°
Already experienced	2,240	1.8		**1.51 (1.02–2.24)**	**2.09 (1.39–3.13)**

^#^ Video slot machines include: Amusement With Prize (AWPs) machines, Video Lottery Terminals (VLTs) and online slot machines

* Estimated through logistic regression models adjusted by year of interview (2018, 2022), gender (male, female), age (continuous), nationality (Italian, not Italian born in Italy, not Italian born abroad), and type of school attended (lyceum, technical college, vocational college). Estimates in bold are statistically significant at 0.05 level

° Reference category

As a supplementary analysis, Supplementary Table 1 shows the distribution of participants according to their knowledge about gambling accessibility and awareness about gambling-related risks over the two surveys. In the 2022 survey, the percentages of participants who declared knowing about bars, tobacco shops, and slots or betting halls where minors were allowed to gamble was significantly lower compared to the 2018 survey (43.8% vs. 56.2%, 38.8% vs. 61.3%, and 20.8% vs. 79.2%, respectively, *p*-value < 0.0001). The percentage of participants who claimed to know of websites that allowed minors to gamble was around 50% in both the survey rounds (*p*-value: 0.1915). Participants aware of the gambling addictive potential accounted for 13.4% of the 2018 sample and 16.5% of the 2022 sample (*p*-value < 0.01).

## Discussion

Two large surveys were conducted among Pavia’s high school students in 2018 and 2022, reporting data on almost 8,000 individuals aged 15–17. Overall, nearly 13% of participants reported lifetime VSM use. Lifetime VSM use exceeded 15% in 2018 and stood at 12% in 2022. Current regular VSMs use was over 1%, in both surveys. Both lifetime and current regular VSM use were more frequent in students with lower school performance and in those with parents or siblings gambling on a daily basis. Lifetime experience of VSM use was more frequently reported by those who had already experienced other risky behaviors, including tobacco smoking, alcohol drinking, psychotropic substance use, sexting, and voluntary self-injury, than in those who did not. Current regular use of VSM was directly associated with a tendency to smoke tobacco, drink alcohol, and use psychotropic substances on a monthly basis or more frequently.

Involvement in gambling in the early years of life can have detrimental effects on health with enduring consequences into adulthood (Armitage, 2021; Messerlian et al., 2005). Hence, the implementation of preventive measures specifically targeting younger individuals is of paramount importance (Calado et al., 2017; Emond & Griffiths, 2020; Floros, 2018). In Italy, In 2012, a legislative decree known as the “Decreto Balduzzi” prohibited the establishment of new slot halls and the installation of additional AWPs within a secure distance around designated “sensitive locations”, including schools, youth gathering places, hospitals, and places of worship (Tavazzani et al., 2020). In 2020, a national law imposed further restrictions on gambling, stipulating, among other provisions, the mandatory inclusion of a health card-based age verification system in all VLTs (Tavazzani et al., 2020). However, the efficacy of such preventive measures in mitigating the burden deriving from these electronic gambling machines has been called into question (Marionneau et al., 2022). In fact, despite scant data on AWP and VLT use among those under 18 years of age, the use of these formats seems still widespread among Italian adolescents: according to the European School Survey Project on Alcohol and Other Drugs (ESPAD), more than 14% of students aged 15–19 had used EGMs during 2022 (*Relazione al parlamento sul fenomeno delle tossicodipendenze in Italia*, 2023). A significant concern is that while regulatory measures might have curtailed minors’ access to VLTs, AWPs, which share many of the addictive features of VLTs, remain exempt from the requirement for age verification using health cards (“DECRETO-LEGGE 28 gennaio, 2019, n. 4 - Disposizioni urgenti in materia di reddito di cittadinanza e di pensioni,” 2019), thereby continuing to allow easy access to children and young adolescents. The landscape is further complicated by Italy’s decision to include VSMs within the permissible offerings of its online gambling platforms, in contrast to the more restrictive approaches adopted by other countries (Casu et al., 2023; Perrot et al., 2022). This creates an additional avenue for accessing VSMs, which is particularly concerning given the reported ease of circumventing security measures on these platforms (Armitage, 2021). Furthermore, the presence of numerous unauthorized websites on the internet that do not require age verification exacerbates the problem (Armitage, 2021; *Libro blu 2021 - Principali performances*, 2021; Montiel et al., 2021).

Despite the inherent limitations of convenience sampling employed in this study, the large sample sizes allow us to suggest a worrying possibility of relatively widespread access to VSMs among underage Italian students. Of particular concern is the fact that over one in 100 students reported regular use of this hazardous gambling format. While the partial comparability of data from 2018 to 2022 limits our ability to comment on the observed modest but significant decline in lifetime VSM use prevalence and to determine if it could reflect an impact of the regulatory interventions, we can reasonably assume that the COVID-19 pandemic might have impacted our findings. Indeed, the national lockdown and the subsequent restrictive measures have compelled the closure of gambling halls and other public places hosting AWPs at different time intervals (Lugo et al., 2021; Odone et al., 2020); as a result, there was a noticeable decline in the use of AWPs and VLTs throughout 2020 and 2021 (*Libro blu 2021 - Principali performances*, 2021; Lugo et al., 2021; Quinn et al., 2022).

Beyond considerations regarding the efficacy of regulatory interventions, gambling harm prevention strategies may fall short if they do not address educational aspects. In this regard, it must be noted that in the 2022 survey, approximately 1 in 5 participants was unaware of the addictive potential associated with gambling, a slightly higher proportion compared to 2018. This alarming finding highlights the urgent need to intensify and tailor educational interventions to effectively address adolescents’ understanding of gambling risks.

In line with previous studies, we observed that both lifetime experience and current regular VSM use were positively associated with lifetime experience of tobacco smoking, alcohol drinking, use of cannabis, cocaine, and other psychotropic substances, as well as sexting and voluntary self-harm (Benedan & Monti, 2020; Buja et al., 2017; Emond & Griffiths, 2020; Gómez et al., 2019; Riley et al., 2021; Zhai et al., 2020). Indeed, during adolescence, these behaviors exhibit a convergence of several predisposing factors both at the social and the individual level, with impulsivity and sensation seeking likely playing a significant role (Gassó et al., 2019; Lockwood et al., 2020; Martínez-Loredo et al., 2019; Pisarska & Ostaszewski, 2020). This marked tendency of regular VSM users to adopt multiple risky behaviors simultaneously suggests that merely addressing the issue of gambling in isolation would likely overlook the broader picture of adolescents’ well-being.

As youth gambling is not a result of a single cause but rather an intricate interplay of biological, psychological, social, and environmental determinants (Moreira et al., 2023), attempting to tackle this issue through a singular approach would be insufficient. Multifactorial intervention strategies recognize the interconnectivity of these determinants and aim to comprehensively targeting them. For instance, while parental and sibling influences are pivotal, as indicated by our findings and previous research (Molinaro et al., 2014; Parrado-González et al., 2023), these are just one piece of the puzzle. Concurrently, there are cognitive biases (Goodie et al., 2023) societal norms (Jiang et al., 2023), and emotional vulnerabilities (Buen & Flack, 2022) that play into adolescents’ gambling behaviors. Therefore, preventive interventions must not only address family dynamics but also include cognitive-behavioral approaches to challenge distorted beliefs, educational initiatives to foster informed decision-making, and peer support programs to create a positive influence circle. Considering the ever-evolving landscape of technology and its impact on gambling accessibility, multifactorial strategies become even more essential. With the proliferation of online gambling platforms, virtual reality, and mobile apps (Lopez-Gonzalez et al., 2020; Oberdörfer et al., 2022; Percy et al., 2021), a comprehensive approach must encompass not only traditional avenues but also the digital realm. This involves regulations, public health educational campaigns, and digital monitoring tools to restrict underage access and promote responsible behavior.

## Strengths and Limitations

We acknowledge our study has both strengths and limitations. Although it was based on a non-representative convenience sample, and we cannot infer prevalence data, we were able to assess VSM use and associated factors on almost 8,000 students, thus representing one of the largest studies available on the gambling behaviors of underage students in Italy. Unlike several epidemiological surveys on gambling which gathered data exclusively on land-based VSMs, our study considered the entire category of VSM, providing a more comprehensive assessment of the phenomenon, also accounting for the influence of technological and regulatory advancements and the impact of the COVID-19 pandemic. In addition, while most previous studies have focused on the problem or disordered gamblers, our research considered lifetime experience and current regular use of VSM. This provided valuable insights into the accessibility of this gambling format and broadened our perspective to include all individuals who engage in this risky activity regularly, even before the development of a problem or disordered gambling pattern. Furthermore, even if a frequency of at least once a month gambling does not necessarily imply the presence of a gambling disorder, there is evidence suggesting a strong relationship between frequent use of land-based and online VSM and gambling-related problems (Afifi et al., 2010; Gainsbury et al., 2019). The cross-sectional design prevented the determination of any causal inferences, although it allowed to quantify the relationships between VSM use and associated factors. Moreover, we were able to adjust our estimates for key potential confounders, including socio-demographic and educational characteristics. Last, the study relies on self-reported data, which is limited by social desirability bias, recall bias, and misinterpretation of questions, particularly those about their habits and psychosocial aspects. Still, the anonymity was guaranteed at the time of data collection, which has likely preserved the accuracy of the responses.

## Conclusions

The information gathered within this study complements the data collected from national and international adolescents’ lifestyles surveillance systems, indicating that in Italy, both land-based and online VSMs are still accessible to minors and might be regularly used by a non-negligible number of them. These findings raise concerns regarding the adequacy of current measures aimed at restricting underage access to VSM in the country, especially considering the continuous expansion of online gambling platforms. Further research is crucial to gain a comprehensive understanding of the extent of this phenomenon, and inform the development of appropriate preventive interventions. As VSM users exhibit a propensity to engage in multiple risk behaviors simultaneously, an integrated, multidimensional and multidisciplinary approach in terms of prevention, diagnosis, and treatment may be required to successfully address this complex public health issue.

## Electronic Supplementary Material

Below is the link to the electronic supplementary material.

Supplementary Material 1

## Data Availability

The data that support the findings of this study are available from the corresponding author on reasonable request.
