# Supplementary material for: P.A.V.I.A. Study: Pervasiveness and Associated Factors of Video Slot Machine Use in a Large Sample of Italian Adolescents
Source: J Gambl Stud. 2024 Jul 22;40(4):1887–904. doi: 10.1007/s10899-024-10334-2 (PMC11557643; doi:10.1007/s10899-024-10334-2)
Supplement: Supplementary file 1 — Supplementary Material 1 [file 10899_2024_10334_MOESM1_ESM.pdf]

Supplementary Table 1 Distribution of 7,959 high school students aged 15-17 by perception and beliefs about gambling in 2018 and 2022. Pavia, Lombardy Region, Italy

	**Year**				
	2018		2022		
	**n**	**%**	**n**	**%**	**p-value**
**Knows about bars where minors can gamble**					**<0.0001**
No	1,554	45.8	2,579	56.2	
Yes	1,828	54.2	2,008	43.8	
**Knows about tobacco shops where minors can gamble**					**<0.0001**
No	1,699	50.4	2,809	61.3	
Yes	1,673	49.6	1,778	38.8	
**Knows about slots/betting hall where minors can gamble**					**<0.0001**
No	2,494	74.0	3,631	79.2	
Yes	878	26.0	956	20.8	
**Knows about websites where minors can gamble**					0.1915
No	1,657	49.1	2,322	50.6	
Yes	1,715	50.9	2,265	49.4	
**Believes gambling can be addictive**					**0.0005**
No	451	13.4	758	16.5	
Yes	2,817	83.5	3,685	80.3	
Does not know	104	3.1	144	3.1	

Authors:
Giansanto Mosconi, Paola Bertuccio, Ilaria Albertin, Marcello Esposito, Anna Polgatti, Franco Taverna, Diego Turchinovich, Sara Russo, Silvia Gaggi, Serena Barello, Andrea Amerio, Silvano Gallus, Lorella Cecconami, Simone Feder, Tomaso Vecchi, Anna Odone

Correspondence to:
Anna Odone,
School of Public Health
Department of Public Health, Experimental and Forensic Medicine
University of Pavia,
Via Forlanini 2, 27100 Pavia, Italy
anna.odone@unipv.it
